# Food Sources of Selenium and Its Relationship with Chronic Diseases

**DOI:** 10.3390/nu13051739

**Published:** 2021-05-20

**Authors:** Wenli Hu, Chong Zhao, Hongbo Hu, Shutao Yin

**Affiliations:** College of Food Science and Nutritional Engineering, China Agricultural University, 17 Qinghua East Road, Haidian District, Beijing 100083, China; sy20183061027@cau.edu.cn (W.H.); zhaoch0206@cau.edu.cn (C.Z.); hongbo@cau.edu.cn (H.H.)

**Keywords:** selenium biofortification, chronic diseases, baseline selenium status, methylated selenium compounds

## Abstract

Selenium (Se) is an essential micronutrient for mammals, and its deficiency seriously threatens human health. A series of biofortification strategies have been developed to produce Se-enriched foods for combating Se deficiency. Although there have been some inconsistent results, extensive evidence has suggested that Se supplementation is beneficial for preventing and treating several chronic diseases. Understanding the association between Se and chronic diseases is essential for guiding clinical practice, developing effective public health policies, and ultimately counteracting health issues associated with Se deficiency. The current review will discuss the food sources of Se, biofortification strategies, metabolism and biological activities, clinical disorders and dietary reference intakes, as well as the relationship between Se and health outcomes, especially cardiovascular disease, diabetes, chronic inflammation, cancer, and fertility. Additionally, some concepts were proposed, there is a non-linear U-shaped dose-responsive relationship between Se status and health effects: subjects with a low baseline Se status can benefit from Se supplementation, while Se supplementation in populations with an adequate or high status may potentially increase the risk of some diseases. In addition, at supra-nutritional levels, methylated Se compounds exerted more promising cancer chemo-preventive efficacy in preclinical trials.

## 1. Introduction

Selenium (Se) is essential for the maintained health of mammals, and its deficiency is common and a serious issue worldwide. The World Health Organization (WHO) shows that there are more than 40 countries and regions globally that suffer from Se deficiency [1]. Approximately 51% of the regions in China have soil that is Se deficient [1]. Se deficiency is a serious hazard to human health and prone to various chronic diseases, such as Keshan disease, Kashin-Beck disease, cardiovascular disease (CVD), diabetes, cancer, inflammatory diseases, subfertility, and viral infections. Therefore, the biofortification strategies to produce Se-enriched foods can help overcome Se deficiency and improve human health. Ample existing evidence has suggested that Se compounds have a protective impact against chronic diseases. Several factors affecting the beneficial activities of Se compounds have been identified, including the baseline Se status, the dosage and forms of Se. A better understanding of the relationship between Se and chronic diseases will help develop more precise solutions to combat the health problems caused by Se deficiency.

## 2. Food Sources of Se

### 2.1. The Overview of Se Contents and Forms in Different Foods

According to results of the ANIBES (“Anthropometry, Intake, and Energy Balance in Spain”) study in Spain, the daily Se intake of the whole population is between 14 and 265 µg/day, with a mean level of 75 ± 1 µg/day [2]. Cereals and grains were the main contributors (46.5%) to Se intake, while animal foods provided the second portion of Se. Fish accounted for 16.7%, meat and meat products 14.9%, milk and dairy products 7.2%, and eggs 5%. All these groups provided more than 85% of the Se intake [2]. Finally, ready-to-eat meals, vegetables, pulses, fruits, sugars, sweets, and non-alcoholic beverages contributed to a small part of the dietary Se intake.

Generally, the Se concentrations in the different foods followed this descending order: animal-based foods > vegetables > cereals > fruits. In addition, the Se content in foods depends to a great extent on Se content in the soil where plants and animals grow. The mean Se content in cereals and animal foods, including meat, fish, milk, and eggs, respectively, ranges from 0.0021–2.11 mg/kg and 0.0042–2.46 mg/kg in China [1]. Vegetables contain a relatively small amount of Se, and its contents in the edible parts of different vegetables in China range from 0.0008 to 5.37 mg/kg, with a mean of 0.067 mg/kg [1]. The Se contents in the different vegetables are in the descending sequence: cruciferous vegetables > liliaceous vegetables > legumes > solanaceous vegetables > leafy vegetables. Cruciferous vegetables, garlic, and onions are considered high-Se-accumulating vegetables and can be Se-enriched from <0.5 mg/kg up to 140–300 mg/kg [3]. Brazil nuts rank at the top of ten products containing the largest quantity of Se [4].

**Figure 1:**
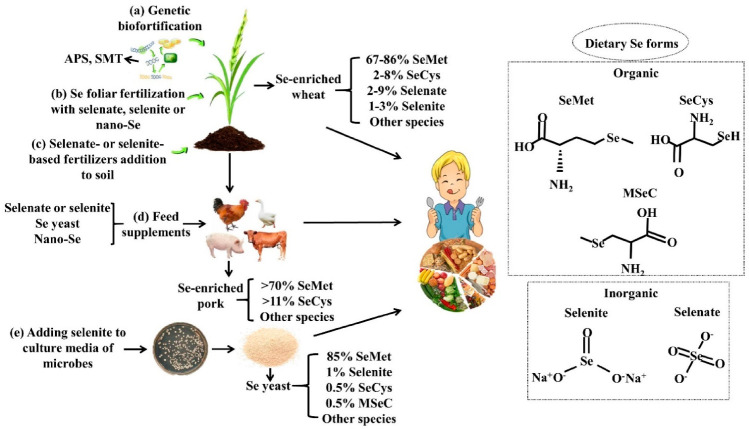
Se biofortification strategies, predominant dietary Se forms, and their percent compositions in Se-enriched foods. Plant-based biofortification mainly consists of (a) genetic biofortification and agronomic biofortification, including (b) and (c). Genetic biofortification approaches include breeding and genetic engineering, which can transfer the Se-enriched genes, such as ATP-sulfurylase (APS) and selenocysteine methyltransferase (SMT), to plants. Different sources of Se are available for feed supplements for domestic animals to produce Se-biofortified animal foods (d), including inorganic (mainly selenite or selenate), organic (mainly Se yeast), and nanoforms of Se; Adding Se, such as selenite, to culture media of microbes (e) to manufacture Se-enriched foods, such as Se yeast.

The predominant dietary Se forms can be divided into inorganic Se, selenate and selenite, and organic Se, selenome-thionine (SeMet), selenocysteine (SeCys) and Se-methylselenocysteine (MSeC). For inatance, MSeC is the main Se form in Se-enriched broccoli, garlic, and onions [5,6]. The predominant species of Se in cereals and bread are SeMet and SeCys [7]. The percent composition of Se species in Se-enriched wheat grains [8], Se-enriched pork [9], and Se yeast has also been identified [10]. The chemical structures of these dietary Se compounds and their percent compositions in Se-enriched foods are summarized in Figure 1.

### 2.2. Se Biofortification

Considering the large-scale Se deficiency in the world, relying on only a few Se-rich regions to achieve the enrichment of natural Se resources, it is unable to meet the demand for Se supplementation. Therefore, people take advantage of a series of biofortification strategies to develop Se-enriched foods. Se biofortification is a biotechnological strategy that increases the Se content in agricultural products by plant breeding, genetic engineering, or agronomic practices [11]. Generally speaking, plant-based biofortification is the most effective and commonly used approach, especially in staple crops. In addition, Se-biofortified animal foods produced by animals fed Se-enriched feed may be another important way to increase dietary Se intake. Microorganisms can also be biological conversion factors for Se enrichment. Se biofortification not only increases the Se content but also enhances the nutritional value of foods. The overview of Se biofortification strategies is shown in Figure 1.

#### 2.2.1. Agronomic Biofortification

Agronomic biofortification is to increase the nutrient (such as Se) concentration in the edible parts of main crops via fertilizers [12]. Agronomic biofortification mainly includes Se addition to soil and Se foliar fertilization, while the fertilizers typically used are selenate- or selenite-based fertilizers. Applied inorganic Se is metabolized to various organic forms by plants, and the structures and amounts depend on the species of plants, and then these plant Se metabolites are consumed by humans and animals. 

In general, selenate (SeVI) and selenite (SeIV) are easily transported through the plant cuticle, and metabolized by the sulfur assimilatory pathway. Firstly, catalyzed by ATP sulfatase and APS reductase, Se (VI) is reduced to Se (IV). Then, Se (IV) can be further converted to selenides (Se-II). Some selenides are metabolized to SeCys by cysteine synthase, which can be transformed into MSeC or SeMet, under the action of Se-methyltransferase or by trans-sulfurylase, respectively [10].

Most studies have shown selenate to be more effective than selenite, which may be because plants absorb more selenate, with the same Se supplementation amount [13]. For example, the total Se content in leek plants was 982 ± 159 mg/kg and 104 ± 33 mg/kg, respectively, grown on selenate and selenite-fertilized soil, showing a 10-fold difference [14]. The total Se concentration in 50 μM selenate and selenite-treated broccoli sprouts was 179 and 98 mg/kg dry weight, respectively, showing an over 1.8-fold difference [15]. Foliar fertilization is more efficient than soil fertilization [16]. For instance, Se content in control lettuce leaves was 46 μg/kg, while treating plants with 100 mg/L Se achieved 784 μg/kg (for soil application), 1708 μg/kg (for foliar application) [17]. Moreover, some beneficial rhizosphere microbes can enhance the soil’s Se phytoavailability [18]. The addition of beneficial rhizosphere microbes to soil might help to improve the Se biofortification of crops.

#### 2.2.2. Genetic Biofortification

Genetic biofortification includes classical breeding and modern genomic approaches. The purpose is to select and develop plant varieties with high Se accumulation capacity according to the difference of Se absorption, which may be related to the differential expression and affinity for Se over S of root sulfate transporters [19,20]. Several genes with positive outcomes for Se biofortification have been targeted by genetic engineering, primarily consisting of sulfate transporters and S-assimilation enzymes, such as ATP-sulfurylase (APS) and selenocysteine methyltransferase (SMT), which is also the key enzyme to form MSeC [21]. The APS transgenics contained 2.5-fold higher shoot Se levels than wild-type Indian mustard [22]. The overexpression of SMT in tobacco plants increased the total Se and MSeC accumulation, and the total Se content in SMT-overexpressing tobacco (~3.8-fold higher) and control plants were 1.87 mg/kg and 0.49 mg/kg, respectively [23].

#### 2.2.3. Se-Biofortified Agricultural Products

Foliar spray and soil application increased the total and organic Se content in cereals. Furthermore, Se-fortified cereals present various nutritional benefits, for example, antioxidants, amino acids, phenols, anthocyanins, and sugars increased [24]. The consumption of Se-biofortified wheat products increased Se intake by 12–35 μg/day, increased glutathione peroxidase activity in the blood, and the concentrations of lipid peroxidation products decreased in the serum of volunteers [25]. Although the statistical significance was not indicated, the risk factors of CVD improved slightly, with the overall cholesterol decreased by 10.3%, triglycerides decreased by 14.5%, and the low-density lipoprotein decreased by 15.1% [25,26]. 

In addition, the researchers also studied the Se fortification of vegetables. Spraying lettuce with Se improved its growth, antioxidant capacity, Se content and yield quality [17]. The application of Se significantly increased the antioxidant capacity, the total phenol, and rosmarinic acid content in basil leaves during harvest [27]. The content of antioxidant flavonoids, naringenin chalcone, and kaempferol increased, and cinnamic acid derivatives decreased in the Se-biofortified tomatoes [28]. Among the crops that can accumulate Se, the Brassicaceae family has received more attention since they are Se-hyperaccumulating plants. Se-fortified broccoli showed higher amounts of phenolic compounds, increased antioxidant and antiproliferative activity, presenting cytocidal activity for a glioma line, especially the seedlings [29]. 

The most commonly used Se biofortification technology in fruits was foliar spray. Spraying with Se enhanced the Se content and the nutritional quality in fruits and their derivates. Fruit Se concentration increased from 0.1 μg/kg to 242 μg/kg when Se was foliar sprayed at 1.5 mg/L, and meanwhile, the antioxidant enzyme activity, the fruit quality, and the storability of apples were also markedly amplified [30]. Se nanoparticles (Se NPs), as a foliar spray, significantly increased the total sugars, phenolic compounds, antioxidants, and anthocyanins in pomegranates [31]. The foliar Se fertilization of olive trees enhanced the Se content and the antioxidant compounds in extra virgin olive oil (EVOO), such as chlorophylls, carotenoids, phenols, and SeMet, which increased the oxidative stability and shelf-life of EVOO [32].

Various experiments have shown that dietary Se supplementation increased the Se concentration in meat and improved the meat quality, such as enhancing glutathione peroxidase activity and the oxidative stability [33], preserving its texture and sensory characteristics [34], altering the lipid metabolism, and decreasing the cholesterol content [35].

Se-enriched foods that rely on microorganisms to transform and produce Se elements include Se-enriched yeast, Se-enriched edible fungi, and Se-enriched probiotics, which are prepared by adding inorganic Se additives, such as sodium selenite, to their corresponding media. In addition, Se-enriched yeast and Se-enriched probiotics can be used for manufacturing food products such as beer, yogurt, or cheese.

### 2.3. Se Nutritional Fortifiers and Se Fortified Foods

In addition to Se in natural foods, Se can be also used as nutritional food fortifiers in formulating milk powder, rice, and its products, wheat flour and its products, cereal flour and its products, bread, biscuits, and milk beverages. The approved forms are sodium selenite, sodium selenate, selenoprotein, Se-enriched edible fungus powder, MSeC, selenized carrageenan, and Se yeast. There are strict requirements for additive amounts; for example, the United States Food and Drug Administration (FDA) recommends that the Se level in infant formula is 2–7 μg/100 kcal [36]. 

## 3. Se Nutritional Status Assessment, Metabolism, Bioavailability and Biological Functions

It is a challenging task to evaluate the Se nutritional status. Se exists in multiple locations of the body, including blood, hair, and nails. Although the Se content in the blood is used as a major biomarker, it only represents short-term exposure to Se [37]. Toenail Se content can reflect long-term external exposures, and compared with fingernails and hair, the possibility of exposure to external contamination is smaller [38]. Therefore, toenails have more potential for assessing Se’s nutritional status in epidemiologic studies of Se and chronic diseases than other biomarkers. 

Se content in foods does not represent the amount available to organisms, and the absorption of Se from foods depends on its bioavailability. The chemical form is a vital factor affecting Se bioavailability. Generally, organic Se compounds are more bioavailable for animals and humans than inorganic species. As for inorganic Se, selenite is more largely transformed into organic metabolites than selenate [39]. SeCys and MSeC are more easily digested by the gastrointestinal tract than SeMet [40]. Moreover, Se in plant foods is more bioavailable than Se in animal foods [41].

**Figure 2:**
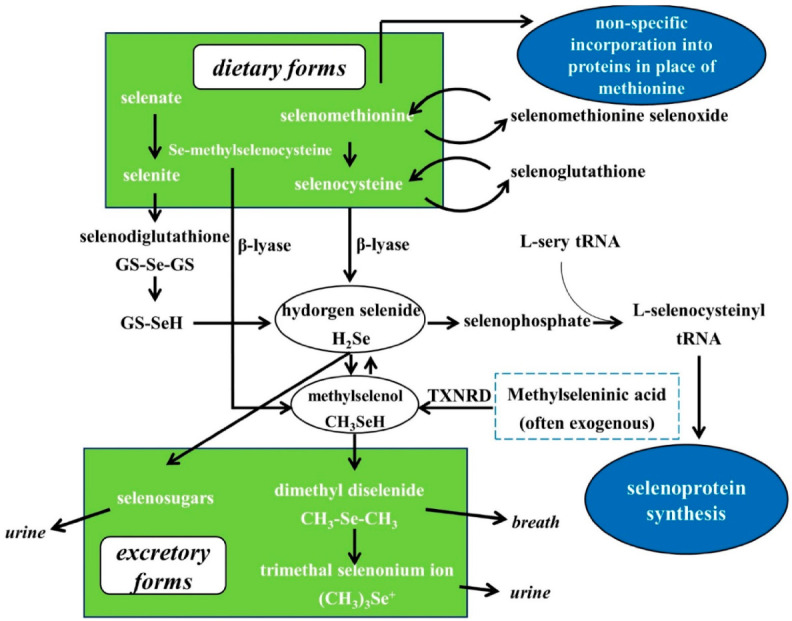
Se metabolism. Most dietary Se can be metabolized to H_2_Se, further involved in the synthesis of selenoproteins and methylated excretion. Methylselenol is a critical Se metabolite for anticancer activity. Se-methylselenocysteine and synthetic methylseleninic acid can be directly converted into methylselenol and bypass the H_2_Se pool. Based on Nicastro and Dunn, 2013 [42].

The metabolism of Se in the human organism is shown in Figure 2. The predominant Se species in food can be divided into inorganic Se, selenate, and selenite, and organic Se, including SeMet and SeCys. All these forms of Se can be metabolized to hydrogen selenide (H_2_Se), which is involved in the selenoprotein synthesis and methylation excretion of Se [4,42,43]. SeMet can participate in synthesizing general proteins instead of methionine or being converted into SeCys via trans-sulfurization. SeCys can be transformed into H_2_Se by β-lyase. Inorganic Se can be converted to H_2_Se through reductive metabolism. H_2_Se can be converted into Selenocysteinyl-tRNA, a crucial transport RNA, to synthesize selenoproteins. When the intake of Se exceeds the need for selenoprotein synthesis (higher than nutritional requirements), H_2_Se is methylated to methylselenol, a key anti-cancer metabolite. With higher intake levels, methylselenol is methylated to dimethylselenide and trimethylselonium ion, which are excreted via respiration and urine, respectively. H_2_Se can also be converted into selenosugars for excretion via urine. Different from the Se compounds mentioned above, MSeC can be directly metabolized into methylselenol by β-lyase [4]. Exogenous methylseleninic acid (MSeA) can be directly reduced by thioredoxin reductase (TXNRD) to methylselenol. Therefore, at supra-nutritional levels (higher than nutritional requirements), MSeC and MSeA are more promising anti-cancer agents.

Se exerts various biological functions primarily via selenoproteins, especially selenoenzymes, such as regulating thyroid hormone metabolism, antioxidant system, oxidative metabolism, and immune system. The antioxidant properties of selenoproteins are mainly due to some selenoenzymes, such as glutathione peroxidases (GPXs), which catalyze reducing hydrogen peroxide, phospholipid peroxides, and lipid peroxides into harmless water and alcohols, protecting cells from oxidation damage. SeCys is considered the 21st amino acid participating in ribosome-mediated protein synthesis, and it is also an integral part of selenoprotein activity. The UGA codon mediates the specific incorporation of SeCys into selenoproteins [44]. Currently, about 25 selenoproteins have been found in mammals and humans [45]. Of these, the functions of some are clearly characterized, such as GPXs, TXNRDs, iodothyronine deiodinases (DIOs), methionine sulfoxide reductase B1 (MSRB1), and selenophosphate synthetase 2 (SEPHS2). The functionality of some non-enzyme members is also gradually better understood [46]. Table 1 lists the mammalian selenoproteins, tissue distribution, and localization, as well as their functions. The selenoproteins are designated according to the official nomenclature [47].

## 4. Chronic Diseases

### 4.1. Cardiovascular Disease

CVD is currently the most prominent causative factor for human mortality and the greatest threat to human health worldwide. The earliest research on the role of Se in the cardiovascular (CV) system can be traced back to Keshan disease, a type of congestive cardiomyopathy that occurred in regions in China suffering from Se deficiency before 1980, and can be entirely prevented by sodium selenite supplementation [48]. A series of prospective observational studies showed a possible non-linear, U-shaped relationship between the baseline Se status and CVD incidence. Within a narrow blood Se range of 55–145 μg/L [49,50], the Se concentration exhibited a significant negative association with CVD risk. Several meta-analyses of previous randomized controlled trials (RCTs) demonstrated that Se supplementation was not effective on CVD prevention [50,51].

However, some evidence showed that Se supplementation plays a positive role in CVD prevention. One randomized controlled trial showed that the baseline Se status in UK pregnant women was relatively low, increasing the risk of pregnancy-induced hypertension, while Se treatment as selenized yeast (60 μg/day) greatly reduced the risk of pre-eclampsia and pregnancy-induced hypertension [52]. According to another study on Swedish elderly citizens, long-term supplementation with Se yeast (200 μg/day) and coenzyme Q10 reduced CV mortality and increased cardiac function [53]. Subsequent analysis of whether the functions of Se and coenzyme Q10 supplementation depends on the baseline Se status showed that supplementation played a role in protecting the heart in people with low baseline Se levels (≤85 μg/L) [54]. Possible related mechanisms involved in the protective effects of Se on the CV system include reduced oxidative stress and inflammation [55,56]. Additionally, plenty of laboratory studies suggested that optimal Se intake could prevent atherosclerosis, the pathological basis of CVD, by reducing oxidative stress, infection, endothelial dysfunction, vascular cell apoptosis, and vascular calcification [48]. Selenoproteins may be related to the prevention of arteriosclerosis, including GPX1, GPX3, GPX4, TXNRD, SELENOP, and SELENOS [48].

In summary, the results of randomized controlled experiments so far are inconsistent, and the protective effect of Se on CVD is still inconclusive, but it was found that subjects with low baseline Se concentrations could benefit from Se supplementation. To determine whether Se is beneficial for CVD prevention, larger and more extensive clinical trials are needed. Some factors, such as the dose and forms of Se, the baseline Se status, and the selenoprotein genotype of the target population [48], should be considered when designing a prevention strategy.

### 4.2. Metabolic Diseases

#### 4.2.1. Diabetes Mellitus

Diabetes mellitus (DM) has become one of the major challenges of public health in the 21st century. Type 2 diabetes mellitus (T2DM) is the most common form of DM, accounting for 90%-95% of DM cases [57]. Indeed, in the 1990s, selenate exhibited anti-diabetic and insulin-mimetic effects in vivo and in vitro studies [58]. However, later observational studies and RCTs showed that the relationship between Se and T2DM is highly complex. The role of Se in preventing T2DM is still inconclusive and is limited to very few human studies.

A meta-analysis based on previous observational studies found a U-shaped non-linear dose-responsive relationship between serum Se and T2DM. Therefore, in individuals with low (<97.5 μg/L) and high serum Se contents (>132.50 μg/L), it was found that there was a positive correlation between serum Se and T2DM [59]. These results indicated that Se deficiency and Se excess are potential risk factors in the development of T2DM. The Nutritional Prevention of Cancer trial (NPCT) showed that Se yeast supplementation (200 μg/day) increased the incidence of T2DM in subjects with the highest baseline Se levels (>121.6 ng/mL) [60]. The Se and Vitamin E Cancer Prevention Trial (SELECT) also found that Se increased T2DM risk, although this was statistically nonsignificant [61]. It should be noted that the median baseline plasma Se level in SELECT (136 μg/L) was higher than in the NPCT (113 μg/L) [43]. Furthermore, these were generally cancer trials in which T2DM was only a secondary endpoint. The synthesis of results from several RCTs revealed that Se supplementation at a low Se status appears to have no adverse effects, while Se supplementation in well-nourished populations may potentially increase the risk of T2DM [62].

#### 4.2.2. Thyroid Diseases

The thyroid gland contains the highest amount of Se among all tissues. Thyroid tissues express a number of selenoproteins such as GPXS, TXNRDS, and DIOS, which play an important role in thyroid hormone metabolism and anti-oxidative stress.

A cross-sectional observational study found that the prevalence of thyroid diseases (hypothyroidism, subclinical hypothyroidism, autoimmune thyroiditis and enlarged thyroid) in Se-deficient areas was significantly higher than that in Se-rich areas [63]. Several studies have already demonstrated the benefits of Se supplementation on autoimmune thyroid disorders. A systematic review and meta-analysis of 16 controlled trials showed that Se supplementation significantly reduced thyroid autoantibody levels in patients with chronic autoimmune thyroiditis [64]. The presence of thyroid autoantibodies is relatively high in women of childbearing age, and pregnant women positive for thyroid peroxidase antibodies are prone to develop postpartum thyroid dysfunction (PPTD) and permanent hypothyroidism [65]. A prospective, randomized, placebo-controlled study suggested that SeMet supplementation (200 μg/day) during pregnancy and in the postpartum period reduced the incidence of PPTD and hypothyroidism [66]. A recent multicenter, randomized, double-blind, placebo-controlled trial also demonstrated that SeMet supplementation (83 μg/day) during pregnancy and after delivery reduced autoantibody titer during pregnancy and postpartum thyroiditis recurrence [67]. Se is also effective in Graves’ disease; Se administration significantly improved quality of life, reduced ocular involvement, and slowed the progression of the disease in patients with mild Graves’ orbitopathy [68]. Despite recommendations only extending to patients with Graves ophthalmopathy, Se supplementation is widely used by clinicians for other thyroid phenotypes. More solid clinical evidence is awaited to determine the role of Se in thyroid disorders. Ongoing and future trials might help identify individuals who can benefit from Se supplementation, based, for instance, on individual Se status or genetic profile [69].

### 4.3. Chronic/Acute Inflammations

Epidemiological data suggest that Se deficiency is positively related to the prevalence of atherosclerosis, rheumatoid arthritis, and viral infections, including HIV/AIDS, and chronic inflammation is the main cause of the disease. Se supplementation in patients with these chronic disorders improved their health status and quality of life [70]. Epidemiological studies have shown that there is an inverse relationship between Se levels and inflammatory bowel disease (IBD), including Crohn’s disease and ulcerative colitis, which can be transformed into colon cancer. Furthermore, laboratory studies have also demonstrated that dietary Se alleviated gastrointestinal inflammation and restored epithelial barrier integrity [71]. Se supplementation increased body weight, colon length, and the survival of mice after treatment with dextran sodium sulfate (DSS) and decreased colitis-associated inflammation [72]. In addition, dietary Se protected against chronic inflammation-induced colon cancer (CICC) in preclinical animal models [73]. Significant associations between the Se status and incidence or severity of asthma have not been consistently demonstrated in human studies. As with the epidemiological data, the results of intervention studies aimed at determining the efficacy of Se supplementation in reducing the incidence or severity of asthma has also been unclear [74]. However, mouse models for asthma have provided more definitive results suggesting that the benefits of Se supplementation may depend on the initial Se status of individuals [75].

Se deficiency has been associated with the pathogenicity of several viruses. In addition, several selenoproteins, including GPXs and TXNRDs, seem to play an important role in different virus replication patterns. Finally, the Se nutritional status of the host may also lead to the transformation of the virus genome from benign or low pathogenic to high virulent [76]. When Se-deficient virus-infected hosts were supplemented with dietary Se, the viral mutation rates diminished, and immune competence to combat viral infections was enhanced [77], which has been proved for at least influenza virus type A and Coxsackievirus B3 (CVB3), and HIV/AIDS [78].

The novel coronavirus infection (COVID-19) seriously threatens human health globally. Recent studies have revealed the potential role of Se in COVID-19 prevention and treatment. A Se deficiency is evident in COVID-19 patients with acute respiratory tract infections [79]. Se levels are associated with mortality risk or cure rate from COVID-19. A cross-sectional study of COVID-19 patients conducted in Germany showed that the serum level of Se was significantly higher in samples from surviving COVID-19 patients as compared with non-survivors [80]. Another retrospective analysis also determined that the recovery rate from COVID-19 had a significantly positive association with hair Se levels in patients in China [81]. The ecological study demonstrated that intake levels of Se are inversely associated with higher COVID-19 incidence and/or mortality [82]. Serum zinc (Zn) and serum Se transporter selenoprotein P concentrations within the reference ranges indicated high survival odds in COVID-19 [83]. Early nutritional interventions with Zn, Se, and Vitamin D might protect against COVID-19 and mitigate the course of COVID-19 [84]. Further clinical trials are required to evaluate the beneficial effects of Se supplementation on COVID-19. In addition, small molecular organic Se compound ebselen exhibited promising anti-COVID-19 activity in vitro experiments [85].

### 4.4. Cancer

#### 4.4.1. Human Studies on Se and Cancer

- Epidemiological studies on Se exposure and cancer risk

Many epidemiological studies analyzed the association between Se exposure and cancer risk, but the results have not been consistent. Several epidemiological studies have shown that there is a negative correlation between Se exposure and risk of some cancer types, but null and direct relations have also been reported. A meta-analysis and meta-regression of RCTs, case-control, and cohort studies found that high serum/plasma Se and toenail Se exposure reduced the risk of breast cancer, lung cancer, esophageal cancer, gastric cancer, and prostate cancer, but it had nothing to do with colorectal cancer, bladder cancer, and skin cancer [86]. Several observational longitudinal studies showed that the risk for site-specific stomach, colorectal, lung, breast, bladder, and prostate cancers was reduced, which was related to the highest Se exposure level compared with the lowest [87]. However, a systematic review of epidemiological studies showed that Se exposure was associated with a possible higher risk of keratinocyte carcinoma [88]. 

Several epidemiological studies demonstrated a non-linear U-shaped dose-responsive association. When plasma/serum Se concentration was between 120 and 160 ng/mL, the risk of some types of cancer, including prostate cancer, was reduced compared with a low plasma Se status, <120 ng/mL. Above 160 ng/mL, the cancer-protective effect is likely to diminish, and the risk of certain types of cancer may increase [3]. Although some observational studies indicated an inverse relationship between Se exposure and the risk of certain types of cancers, they cannot be considered evidence of a causal relationship and display many limitations, including exposure misclassification and unmeasured confounding [89]. Accordingly, RCTs are considered next. 

In addition, hepatocellular carcinoma patients undergoing liver transplantation (LT) displayed a notable Se deficiency, and Se status was higher in survivors than non-survivors. Serum Se status may serve as a prognosis marker of LT, and thus, adjuvant Se supplementation may support convalescence [90].

- Human intervention studies with Se

RCTs assessing the impact of Se supplementation on cancer risk have found inconsistent results. The Linxian General Population Nutrition Intervention Trial (NIT) reported that the total cancer mortality was significantly reduced in adults who received beta carotene, vitamin E, and Se supplementation [91]. The NPCT found that the supplementation of Se as selenized yeast (200 μg/day), which contains mostly SeMet, did not have a significant impact on the incidence of basal cell or squamous cell carcinoma, but led to a significant decline in the total cancer mortality, overall cancer incidence, and incidences of lung, colorectal, and prostate cancers [92]. The NPCT also suggested that the incidence of prostate cancer (PCa) decreased significantly only among the subjects with low serum Se levels (<121.6 ng/mL), and there was no risk reduction in subjects with high serum Se concentrations (>121.6 ng/mL) [93]. 

Following the NPCT, a series of phase III clinical trials against prostate and lung cancer was carried out in North America, including SELECT, SWOG9917 [94], ECOG NBT [95], and ECOG5597 [96]. The primary endpoint of all these trials is cancer incidence, but none of them show the efficacy of SeMet or Se-yeast. In fact, follow-up analyses of SELECT showed that Se supplementation increased the risk of high-level PCa among men with a higher Se status [97]. The Se and Celecoxib (Sel/Cel) Trial found that selenized yeast supplementation (200 μg/day) did not prevent the overall recurrence of colorectal adenoma, but the recurrence rate decreased by 18% among participants with advanced adenoma [98]. 

Major reasons for the failure of these studies were associated with the baseline Se levels of subjects, the dose levels and forms of Se supplementation. The baseline Se levels of subjects for these newer trials were higher than in NPCT, which prevented people from deriving additional benefits from Se supplementation. In addition, cell culture and animal models did not support the dose and forms of Se selected for human clinical trials. In prostate cancer cells, 100–500 µM SeMet was needed to suppress growth and induce apoptosis [99]. Such a high level of oral supplement dose cannot be achieved. SeMet did not have an inhibitory effect against human PCa xenografts [100]. 

In summary, although the results of RCTs so far are inconsistent and the protective effect of Se against cancer is still uncertain, it was revealed that subjects with a low baseline Se status could advantage from Se supplementation. To determine the outcome of Se on cancer prevention, more extensive clinical trials are necessary. The dose and chemical form of Se, the baseline Se level of the subjects, and cancer type/grade are all important factors related to the impact of Se on cancer.

#### 4.4.2. Preclinical Studies on the Anticarcinogenic Effects of Different Forms of Se

A wealth of preclinical data demonstrates that methylated Se compounds, which can directly generate methylselenol, exhibit more efficient anti-cancer activities than other Se compounds that are metabolized through the H_2_Se pool meanwhile lacking the genotoxicity produced by inorganic Se. 

MSeC and MSeA are typical methylated Se compounds. MSeC is directly converted to methylselenol via β-lyase. MSeA is reduced to methylselenol by nonenzymatic and enzymatic processes involving GSH and NADPH [101]. MSeC was more active than selenite or SeMet in tumor inhibition in a chemically induced breast cancer model in rats [102]. MSeA and MSeC exerted dose-dependent inhibition of human PCa xenograft growth, and both were more potent than SeMet and selenite [100]. MSeA significantly reduced the metastatic pulmonary yield of Lewis lung carcinoma (LLC); however, SeMet did not [103]. Furthermore, MSeA inhibited cancer cell growth and induced apoptosis more effectively than MSeC in cell culture models [101]. That may due to the β-lyase present in the intestine, liver, kidney, mammary gland, and other animal tissuses [104], and MSeC may not be metabolized into methylselenol in vitro. 

Se NPs have recently emerged as promising anti-cancer agents. Se NPs significantly inhibited human esophageal cancer xenograft growth via suppressing tumor angiogenesis and activating the immune system [105]. Tellurium-Se nanoparticles almost completely eradicated human hepatocellular carcinoma and lung cancer xenografts [106]. In addition, Se from Se-rich food sources exhibited optimal chemo-preventive efficacy. Se-enriched milk significantly lowered colonic tumor incidence and tumor multiplicity [107]. Se-enriched malt inhibited the angiogenesis of hepatocarcinoma [108].

#### 4.4.3. Possible Mechanisms for Anticarcinogenic Actions of Se

**Figure 3:**
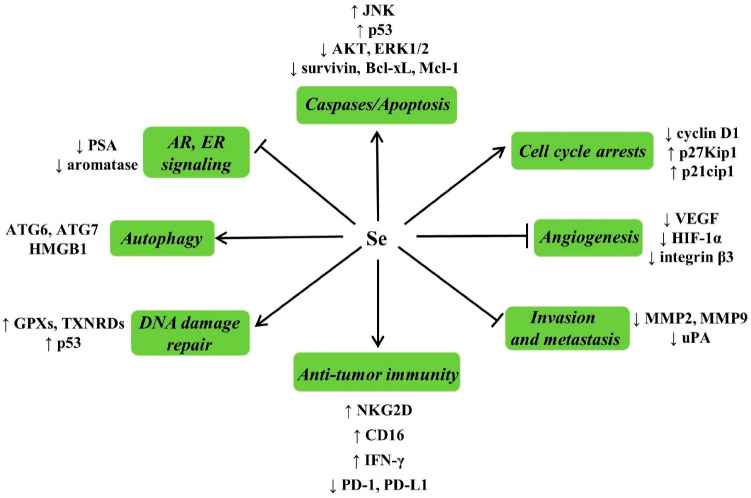
Possible mechanisms of Se against cancer and related molecular targets. Se has been shown to induce apoptosis, cell cycle arrests, inhibit angiogenesis, invasion and metastasis, potentiate anti-tumor immunity, stimulate DNA damage repair, induce autophagic cell death, suppress the androgen receptor (AR), estrogen receptor (ER) signaling, and modulate other processes involved in carcinogenesis.

A better mechanistic understanding of the biochemical effects and molecular targets of Se will provide an in-depth perspective for analyzing the results of clinical trials and designing new trials [109]. Possible mechanisms of the effects of Se against cancer are summarized in Figure 3. 

Apoptosis induction is a mechanism mediating the anticancer activity of Se. MSeA exposure caused caspase-mediated apoptosis in DU145 human PCa cells, which was associated with decreased phosphorylation of Protein Kinase B (AKT) and extracellular regulated kinase1/2 (ERK1/2) [110]. Selenite induced p53 Ser-15 phosphorylation and caspase-mediated apoptosis in LNCaP human PCa cells [111]. MSeA can also enhance the apoptosis induced by chemotherapeutic drugs or biologics in various cancer cell types through inhibition of survival molecules such as survivin, Bcl-xL [112], and Mcl-1 [113,114]. MSeA exposure caused a profound G1 arrest in DU145 cells, which was associated with increased expression of p27kip1 and p21cip1 [110]. The induction of cancer epithelial cell apoptosis and inhibition of cell proliferation by Se in vivo is related to the decreased expression of cyclin D1, increased levels of p27kip1, and c-Jun NH2-terminal kinase (JNK) activation [115].

Angiogenesis is a basic and necessary component of tumor growth, development and metastasis. MSeA reduced the secretion levels of vascular endothelial growth factor (VEGF) in breast cancer cells and inhibited the growth of xenograft [116]. MSeA reduced the metastatic spread of PCa cells to the lungs by downregulating hypoxia-inducible factor-1α (HIF-1α) and its downstream targets VEGF and glucose transporter 1 (GLUT1) [117]. MSeA inhibited angiogenesis not only by down-regulating the expression of integrin β3 but also by disorganizing the clustering of integrin β3 [118]. Matrix metalloproteinase-2 (MMP-2) and matrix metalloproteinase-9 (MMP-9) degrade the extracellular matrix and basement membrane [119], correlated with tumor invasion and metastasis. The urokinase plasminogen activator (uPA) system plays a role in the invasion and metastasis of cancer cells. Dietary supplementation with MSeA reduced spontaneous metastasis of LLC in male C57BL/6 mice by inhibiting the uPA system and reducing angiogenesis [103,120]. Selenite inhibits the invasion of tumor cells via decreasing expression of MMP-2, MMP-9, and uPA [121].

Se has been found to potentiate anti-tumor immunity. SeNPs enhanced γδ T cell anti-tumor cytotoxicity, and cytotoxicity related molecules including natural killer cell group 2 member D (NKG2D), CD16, and IFN-γ were upregulated, but meanwhile, programmed death protein 1 (PD-1) expression of γδ T cells was downregulated [122]. MSeA sensitized ovarian cancer cells to T-cell mediated killing by decreasing programmed death ligand 1 (PD-L1) and VEGF Levels [123]. Stimulation of DNA damage repair is another key mechanism of the cancer-preventive function of Se. Possible mechanisms by which Se enhance DNA damage repair including increasing the synthesis of antioxidant selenoproteins such as GPXs and TXNRDs [124]. Significantly, p53 can maintain genetic stability through removing DNA-damaged cells and activating DNA repair machinery [125]. SeMet facilitated DNA repair through activating p53 by redox regulation [126].

The androgen receptor (AR) is a vital driver and a common therapeutic target for PCa. MSeA suppressed the AR expression and AR signals to downregulate prostate-specific antigen (PSA) in human PCa cells [127]. The signaling of estrogen receptor (ER) is very important for the development of breast cancer. MSeA has been proven to disrupt ER signaling in human breast cancer cells [128]. In addition, MSeA effectively suppressed aromatase activation in human breast tumor cells [129], which makes it a potential chemopreventive agent for breast cancer in postmenopausal obese women. Autophagy also plays an important role in Se-induced cell death. In malignant tumor cells, selenite induced superoxide-mediated mitochondrial damage and subsequent autophagic cell death, autophagy-related (ATG) proteins, ATG6 and ATG7, involved in this process [130]. Under hypoxic conditions, the reductive stress induced by H_2_Se promoted cell autophagy via regulating the redox of human high-mobility group protein B1 (HMGB1), and excessive autophagy leads to autophagy-associated cell death in human hepatocellular carcinoma HepG2 cells [131]. 

#### 4.4.4. Se and Cancer Adjuvant Therapy

The cancer chemo-preventative effect of Se has been demonstrated in many experimental models. In addition, combining Se with conventional cancer therapy, especially chemotherapy and radiation, has achieved encouraging results in both preclinical studies and a series of human trials. Se has been confirmed to augment the anti-cancer efficacy of chemotherapy and radiation. In addition, some experiments have found that Se supplementation is the potential for protecting against toxicity and the side effects of radiotherapy (RT) and chemotherapy.

- Enhancing antitumor efficacy

Extensive preclinical experiments have shown the therapeutic potential of Se as an apoptotic enhancer of various chemotherapy drugs, including cisplatin [132,133], oxaliplatin [134], irinotecan [135], paclitaxel [112,136], etoposide [136], SN-38 [136], doxorubicin [137], TRAIL [138,139], and ABT-737 [113]. The combined SeMet and ionizing radiation treatment resulted in the increased cell termination of human lung cancer cells [140]. MSeA has also been demonstrated to sensitize head and neck squamous cell carcinoma (HNSCC) to radiation, potentially by inducing lipid peroxidation [141]. Few clinical studies have evaluated the impact of Se supplementation during chemotherapy or radiation on treatment efficacy. Researchers have found that Se supplementation with chemotherapy significantly improved clinical outcomes, including an increased tumor response rate and prolonged overall survival time in patients with non-Hodgkin lymphoma (NHL) [142]. Another multi-center, phase III trial showed that selenite supplementation increased the blood Se levels in Se-deficient patients while reducing the number of episodes and severity of diarrhea cases caused by RT [143]. Although the overall survival rate showed an upward trend, there was no significant change in the disease-free survival at a median follow-up of 67 months [144].

- Reduction in toxicity

Extensive preclinical data have demonstrated that various Se compounds reduced the toxicity of radiation, as well as the organ-specific toxicity of multiple chemotherapy agents. MSeC provided great protection against organ-specific toxicity induced by clinical chemotherapeutics in nude mice, which included diarrhea, stomatitis, alopecia, bladder, kidney, and bone marrow toxicities [135]. MSeA protected normal cells from cytosine arabinoside or doxorubicin chemotherapy and radiation toxicity while enhancing their therapeutic effects against malignant cells [145]. Human studies also indicated that Se supplementation reduced the risk of side effects from chemotherapy and RT. Two randomized phase III clinical studies showed that adjuvant Se supplementation successfully decreased RT-induced diarrhea in patients with carcinomas of the uterus and prevented the ageusia and dysphagia due to RT in patients with head and neck cancer [146]. Supplementation with Se also reduced the side effects of chemotherapy in cancer patients, especially by improving the conditions of patients with fatigue, nausea, and poor physical performance, and improving the function of kidney and liver [147]. However, the potential beneficial effects of adjuvant Se supplementation on cancer therapy may depend on the Se dosage and species, as well as the type of treatment and cancer. For example, in cancer patients treated with irinotecan, large superdoses (>2000 μg/day) of SeMet to increase the plasma Se to >15–20 μM did not seem to provide any additional benefits to the patient and did not decrease the toxicity of the treatment [148].

### 4.5. Fertility

Observations from previous studies (both animal and human) show that Se is essential for spermatogenesis and male fertility. In terms of the potential molecular mechanisms, Se plays a structural role in sperm and has bearings on sperm motility, chromatin integrity, and fertility rates. In addition, the adequate transport of Se for the synthesis of certain selenoproteins in the testes is vital for proper spermatogenesis and steroid biosynthesis [149]. Sodium selenite treatment can prevent adult male Wistar rats from testicular damage induced by varicocele [150]. It can be clearly seen from previous studies (both animal and human) that Se is essential for optimal reproduction in females [151]. One multi-center prospective cohort study found that lower maternal plasma concentrations of Se were associated with longer pregnancy and lower birth rate [152]. The exact molecular mechanisms through which Se modulate female reproduction is still unclear.

## 5. Clinical Disorders and Dietary Reference Intakes

The intake range between Se deficiency and toxicity is relatively narrow. It is recommended that the minimum intake is 40 μg/day. Deficiency symptoms, including immunity loss, viral infections, and reproductive barriers, are obvious when the intake is less than 11 μg/day [153]. In total, 100–200 μg Se per day is needed to reduce genetic damage and cancer progression for humans [153]. The clinical features of Se toxicity, or selenosis, including hair and nail brittleness and loss, gastrointestinal disturbances, skin rash, garlic breath, fatigue, irritability, keratosis, rickets, and nervous system disorders, appeared at a Se intake of 900 μg/day [154]. 

The Se concentration in the environment has aroused global concern, with the main environmental pollutant Se forms being SeO_4_^2−^ and SeO_3_^2−^ due to high water solubility. It has long been known that Se was capable of antagonizing mercury (Hg) toxicity. Recently, Hg also was reported to display detoxification towards highly toxic dosages of Se [155]. A porous polymer network designed based on Hg/Se antagonism detoxification mechanism almost completely removed toxic anions (SeO_4_^2−^ and SeO_3_^2−^) and metals (Hg^2+^) in water [156]. Microbial reduction played a crucial role in the detoxification of Se excess. Extracellular polymeric substances reduced selenite to insoluble and less toxic elemental Se and enhanced microbial detoxification towards selenite in water [157]. A rhizosphere microbe, *Azospirillum brasilense,* was able to efficiently reduce toxic selenite to Se^0^S^0^-nanoparticles, which may contribute to decreasing Se toxicity in soil and water [158].

Dietary reference intakes (DRIs) are a set of reference values for evaluating whether the dietary nutrient supply meets human needs, whether there is a risk of excessive intake and if it is beneficial in preventing certain chronic diseases. It usually includes estimated average requirement (EAR), referring to the nutritional amount that will maintain a specific biochemical or physiological function in half the people of a given age and sex group; recommended nutrient intake (RNI), which is the average daily amount of a nutrient considered enough to meet the known nutritional needs of almost all healthy people and a goal for dietary intake for individuals; adequate intake (AI), which is the average amount of a nutrient that seems to be sufficient to maintain a certain level and a value used as a guide for nutrient intake when an RNI cannot be determined; and tolerable upper intake level (UL), that is, the maximum nutrient intake that appears safe for most healthy people and beyond which there is an increased risk of adverse health effects [159]. 

Different countries or organizations set different DRIs. WHO/FAO set the RNI of Se for adults as 26–34 μg/day [1]. The RNI of Se for adults is set at 55 μg/day in the USA and Canada [160]. The Chinese Nutrition Society recommended 60 μg Se intake daily for adults [161]. The UL for Se through diet or supplements for adults is set at 400 μg/day [70]. DRIs are also different for various age and sex groups. For example, in China, the RNI of Se for children is 25–55 μg/day, for teenagers, adults, and the old is 60 μg/day, in addition, for pregnant women is 65 μg/day and for lactating women is 78 μg/day. Se tolerance varies among people of different ages; the UL of Se for children is 100–300 μg/day, for teenagers is 350 μg/day, and for adults is 400 μg/day. Moreover, the recommended intake levels of Se are 75 µg for men and 60 µg for women per day in the UK [3].

## 6. Conclusions and Perspectives

Ample evidence exists suggesting that Se has a protective effect on the CV system, diabetes, some types of cancer, inflammatory diseases, viral infection, and subfertility. However, the relationship between Se and human health is complex, as exemplified by the observation that the effects of Se supplementation trials are dependent on baseline Se status, the dose and forms of Se. There is a U-shaped non-linear dose-responsive relationship between Se status and health effects. Subjects with a low baseline Se status could benefit from Se supplementation, while those with an adequate or high status might be affected adversely. In terms of epidemiological studies, toenails are more desirable as a biomarker of the Se status. In addition, at supra-nutritional levels, the methylated forms of Se exerted more promising cancer chemo-preventive activities in preclinical trials. To define more precise relationships between Se and health effects, more clinical and preclinical trials are necessary. The following issues need to be addressed in the future:

- The baseline Se range suitable for Se supplementation still needs to be defined;
- The accurate markers for the assessment of Se status remain to be established;
- We should pay more attention to the relationship between toenail Se and chronic diseases in the future;
- How to enrich the methylated forms of Se in foods is a direction worth exploring;
- The anti-cancer activities of methylated Se compounds remain to be investigated in clinical studies;
- Novel mechanisms for anticarcinogenic actions of Se need to be further explored, and the key mechanisms remain to be identified.

## Figures and Tables

**Table 1:** Mammalian selenoproteins with characterized functions. Based on Labunskyy et al., 2014; Davis et al., 2012; Avery and Hoffmann, 2018; Gladyshev et al., 2016 [44,45,46,47].

Selenoprotein (Abbreviation)	Tissue Distribution ^a^	Localization	Functions
Glutathione peroxidase 1 (GPX1)	Blood, kidney, liver, placenta	Cytosol	Reduces cellular H_2_O_2_ and lipid peroxides
Glutathione peroxidase 2 (GPX2)	Gastrointestinal tract, liver, mammary	Cytosol	Reduces peroxide in the gut
Glutathione peroxidase 3 (GPX3)	Epididymis, kidney, plasma	Plasma	Reduces peroxide in blood
Glutathione peroxidase 4 (GPX4)	Liver, testis	Cytosol; mitochondria; nucleus (testis-specific)	Reduces phospholipid peroxide
Glutathione peroxidase 6 (GPX6)	Embryos, olfactory epithelium	Cytosol	Reduces cellular H_2_O_2_ in the olfactory epithelium
Thioredoxin reductase 1 (TXNRD1)	Heart, kidney, liver	Cytosol	Regenerates reduced thioredoxin
Thioredoxin reductase 2 (TXNRD2)	Adrenal gland, heart, kidney, liver	Cytosol	Catalyzes a variety of reactions, specific for thioredoxin and glutaredoxin systems
Thioredoxin reductase 3 (TXNRD3)	Testis, heart, kidney, liver	Mitochondria	Reduces the oxidized form of thioredoxin and glutaredoxin 2
Iodothyronine deiodinase 1 (DIO1)	Kidney, liver, thyroid	Plasma membrane	Important for systemic active thyroid hormone levels
Iodothyronine deiodinase 2 (DIO2)	Brain, brown adipose tissue, pituitary	Endothelial reticulum	Important for local active thyroid hormone levels
Iodothyronine deiodinase 3 (DIO3)	Brain, placenta, skin	Plasma membrane	Inactivates thyroid hormone
Methionine sulfoxide reductase B1 (MSRB1)	Liver, kidney	Cytosol	Reduces methionine- R-sulfoxide residues in proteins to methionine
Selenophosphate synthetase 2 (SEPHS2)	Kidney, liver, testis	Cytosol	Synthesis of selenophosphate
Selenoprotein F (SELENOF)	Liver, prostate	Endoplasmic reticulum (ER)	Involved in protein folding
Selenoprotein H (SELENOH)	Unknown ^b^	Nucleus	Involved in redox sensing and transcription
Selenoprotein I (SELENOI)	Unknown ^b^	Membrane	Involved in phospholipid biosynthesis
Selenoprotein K (SELENOK)	Unknown ^b^	ER membrane	Modulates Ca^2+^ influx that affects immune cell function; component of ER-associated degradation
Selenoprotein M (SELENOM)	Brain	ER	Protein folding in ER
Selenoprotein N (SELENON)	Brain, heart, liver, muscle	ER membrane	Proper muscle development
Selenoprotein O (SELENOO)	Unknown ^b^	Mitochondria	Unknown ^c^
Selenoprotein P (SELENOP)	Liver, plasma	Plasma	Se transport and antioxidant function
Selenoprotein S (SELENOS)	Unknown ^b^	ER membrane	Involved in ER-associated degradation
Selenoprotein T (SELENOT)	Unknown ^b^	ER and Golgi	Involved in redox regulation and cell anchorage
Selenoprotein V (SELENOV)	Testes	Cytosol	Unknown ^c^
Selenoprotein W (SELENOW)	Brain, muscle, testes	Cytosol	Necessary for muscle function

^a^ Selected rodent and human tissues in which selenoprotein expression is relatively high. ^b^ Protein expression is unknown. However, mRNA has been detected in several tissues. ^c^ Function is unknown. Discovered by in silico analysis.
